# Three-Dimensional Structure of PANI/CdS NRs-SiO_2_ Hydrogel for Photocatalytic Hydrogen Evolution with High Activity and Stability

**DOI:** 10.3390/nano9030427

**Published:** 2019-03-13

**Authors:** Jinrong Lu, Xin Zhang, Huiyuan Gao, Wenquan Cui

**Affiliations:** College of Chemical Engineering, Hebei Key Laboratory for Environment Photocatalytic and Electrocatalytic Materials, North China University of Science and Technology, Tangshan 063210, Hebei, China; lujinrong@ncst.edu.cn (J.L.); 1319023042zx@gmail (X.Z.); hygao@tju.edu.cn (H.G.)

**Keywords:** CdS NRs, polyaniline, SiO_2_ hydrogel, photocatalytic hydrogen evolution

## Abstract

Three-dimensional PANI/CdSNRs-SiO_2_ hydrogel (CdS NRs-PANI-SiO_2_) was synthesized by loading polyaniline (PANI) onto the semiconductor CdS nanorods (NRs) surface and loading the binary complex on SiO_2_ gel. The structure, optical properties, and electrochemical properties of the composite were studied in detail. The hydrogen production amount of CdS NRs-PANI (3%)-SiO_2_ (20%) increased in comparison with CdS NRs and reached 43.25 mmol/g in 3 h under visible light. The three-dimensional structure of SiO_2_ hydrogel increased the specific surface area of the catalyst, which was conducive to exposing more active sites of the catalyst. In addition, the conductive polymer PANI coated on CdS NRs played the role of conductive charge and effectively inhibited the photo-corrosion of CdS NRs. In addition, the recovery experiment showed that the recovery rate of the composite catalyst reached 90% and hydrogen production efficiency remained unchanged after five cycles, indicating that the composite catalyst had excellent stability.

## 1. Introduction

Since the discovery of titanium dioxide for hydrogen generation through water decomposition by Fujishima in 1972 [1], photocatalytic hydrogen production has opened new possibilities for converting low density solar energy into high energy density hydrogen fuel to solve energy crises [2,3,4,5,6]. Today, the development and realization of an efficient and stable photocatalyst system for practical applications is still a major challenge. One-dimensional nanostructures such as nanorods (NRs) and nanowires (NWs) of inorganic semiconductors have been used for hydrogen evolution under illumination. Due to the inherent anisotropic structure, NRs and NWs present many unique properties [7,8,9] such as a large specific surface area, continuous path for electron transmission, and enhanced light absorption and scattering based on the long axis.

Cadmium sulfide (CdS) has been widely exploited as a promising photocatalyst for hydrogen evolution because of its broad-range light absorption and suitable photoredox potential [10]. In addition, its nanostructure of NRs has broad application prospects under visible light irradiation due to its special one-dimensional structure with a large aspect ratio and effective charge transfer [11,12,13,14]. However, past studies revealed that CdS suffers from ultrafast recombination of photoinduced electrons, holes, and serious photo-corrosion [15,16] which obstruct further improvement of its photocatalytic activity. So, it is urgent to develop some strategies for improving the efficiency of charge separation.

Conductive polymers such as PANI (polyaniline), PPY (polypyrrole), and P3HT (polythiophene) possess excellent electrical conductivity, processability, and stability [17], making them suitable for modifying inorganic semiconductors to develop composite photocatalysts [18,19]. For example, previously our group reported on the core–shell structure based on Ag_3_PO_4_ coated with PANI, and the results showed that fabrication of the core–shell structure with conductive polymer was an effective technique for improving the photocatalytic activity and stability of Ag_3_PO_4_ [20]. In addition, we reported that P3HT was coated on the surface of Ag_3_PO_4_ to form a core–shell structure composite [21]. Its photocatalytic performance was improved greatly because the large contact surface could form a special energy band structure and quickly transfer photo generated electrons. PANI is one of the most widely used conductive polymers and has a low bandgap (2.8eV) with strong absorption in visible light [22]. Therefore, PANI would be a good candidate for modifying CdS NRs to produce hydrogen by water decomposition under visible light.

Recently, photocatalysts with three-dimensional (3D) structures attracted much attention because of the large specific surface area, adsorption capacity, exposed active sites, and high stability [23]. Adopting an inorganic hydrogel to fabricate the composite hydrogel is a feasible strategy. Silica (SiO_2_) hydrogel has been widely used in biomimetic and biomedical materials as an environmentally friendly material. Furthermore, it has no absorbance and no shading effect. So, SiO_2_ hydrogel is an excellent candidate for developing a photocatalyst with a network structure [24,25]. In this work, we combined CdS NRs with polyaniline and loaded the binary complex on SiO_2_ gel to obtain a composite (CdS NRs-PANI-SiO_2_) with a micro network structure. The addition of polyaniline enhanced the separation rate of photo generated charges and inhibited the photo corrosion of CdS. Meanwhile, SiO_2_ gel increased the specific surface area of the composite catalyst, which provided more active sites for hydrogen production. Importantly, the composite can be recovered easily because of the three-dimensional SiO_2_ gel structure, which would reduce energy consumption and catalyst recovery costs. The structure, optical properties, and electrochemical properties of the composite were studied in detail. Photocatalytic activity of hydrogen production in the visible light was evaluated in the composite hydrogel. The role of PANI and SiO_2_ gel on improving the activity of CdS NRs is also clarified. The composite hydrogel represented the characteristics of high specific surface area, low cost, and high stability.

## 2. Experimental Section

### 2.1. Materials

Polyaniline (PANI) was obtained from Jilin Zhengji Corp. SiO_2_ was from Tianjin North Tianyi Chemical Reagent Factory (Tianjin, China). Ethylenediamine was purchased from Tianjin Beilian Fine Chemicals Development Co., Ltd. (Tianjin, China). Thiourea and cadmium nitrate were purchased from Tianjin Yongda Chemical Reagent Co., Ltd. (Tianjin, China). Chloroplatinic acid and Lactic acid were purchased from Tianjin Guangfu Fine Chemical Factory Co., Ltd. (Tianjin, China). All chemicals were analytical purity and used as received.

### 2.2. Synthesis of CdS NRs

CdS NRs was prepared using the solvothermal method reported in the literature [26]. In general, 1.067 g of cadmium nitratetetrahydrate and 1.316 g of thiourea were dissolved in ethylenediamine (80 mL) under stirring to form a uniform mixture. The solution was then stirred for 1h and transferred to a Teflon-lined stainless-steel autoclave (100 mL). The autoclave was heated to 180 °C and maintained for 24 h. After cooling to room temperature, the sediment was collected by centrifugation and washed several times with anhydrous ethanol and deionized water. Finally, the product was dried for 12 h in a vacuum oven at 60 °C.

### 2.3. Synthesis of Composites CdS NRs-PANI

A certain amount of PANI was added into 20 mL of deionized water followed by sonication for 8 h until the PANI was evenly scattered in the water. Then 0.2 g CdS NRs was added directly to the PANI solution until a uniform and stable solution was formed. The resulting solution was vacuumed and dried at 60 °C. CdS NRs-PANI (3%) was represented by a quality ratio of 3% PANI and 97% CdS NRs.

### 2.4. Synthesis of CdS NRs-SiO_2_Hydrogel

The hydrogels of CdS NRs-SiO_2_ were prepared by an alkali solution and acid coagulation method [27]. A certain proportion of SiO_2_ and CdS NRs were put into 3 mL of NaOH (5 mol·L^−1^) solution and dispersed by ultrasound for 30 min. The mixed hydrogel of CdS NRs-SiO_2_ was formed by adding HCl (3 mol·L^−1^) to adjust the pH of the mixture to 4–7. The mixed hydrogel of CdS NRs-SiO_2_ was used after freeze-drying. CdS NRs-SiO_2_ (20%) was represented by aquality ratio of 20% SiO_2_ and 80% CdS NRs.

### 2.5. Synthesis of Composites CdS NRs-PANI-SiO_2_

A certain proportion of SiO_2_ and CdS NRs-PANI composite were added into 3 mL NaOH solution (5 mol·L^−1^) and were dispersed under sonication for 30 min. HCl (3 mol·L^−1^) was added into the solution drop by drop under stirring, and the pH of the mixture was adjusted to 4–7 to form CdS NRs-PANI-SiO_2_ mixed hydrogel. Then the hydrogel was freeze-dried to obtain the photocatalyst solid. As an example of the composite’s name, CdS NRs-PANI (3%)-SiO_2_ (20%) was represented by 20% SiO_2_ and 3% PANI.

### 2.6. Photocatalytic Hydrogen Production Activity

The photocatalytic hydrogen production experiment was performed in a PRG–801 photoreactor. A 500 W xenon lamp (320–780 nm, 80 mW/cm^2^) attached to the photocatalytic performance evaluation system (Suncat instruments PGS-15, Beijing, China) was used to irradiate the bottom of the reactor. The 15 mg of catalyst was dispersed in 30 mL deionized water, 10 wt % lactic acid was added as sacrificial agent, and 2 mL H_2_PtCl_6_ (0.1 mg/mL) was added into the suspension. The amount of hydrogen produced by irradiation was monitored by Shimadzu Gas Chromatography (GC-2014 C). The apparent quantum efficiency (AQE) was calculated according to the following equation:

(1) $$\begin{array}{l} \mathrm{AQE}=\frac{\mathrm{Number}\;\mathrm{of}\;\mathrm{reacted}\;\mathrm{electon}}{\mathrm{Number}\;\mathrm{of}\;\mathrm{incident}\;\mathrm{photons}}\times 100\%\\ \;\;\;\;\;\;=\frac{{\mathrm{Number}\;\mathrm{of}\;\mathrm{eloved}\;H}_{2}\;\mathrm{molecules}*2}{\mathrm{Number}\;\mathrm{of}\;\mathrm{incident}\;\mathrm{photons}}\times 100\% \end{array}$$

Each cycle of hydrogen production lasted 3 h in the stability test. After one cycle the hydrogen obtained in the reactor was removed by vacuum extraction with argon, and the mixed solution is replaced by new lactic acid. All the reaction conditions in the long-term stability test are the same as those in the photocatalytic hydrogen production mentioned above.

### 2.7. Characterization

The X-ray diffraction (XRD) was tested by a D MAX 2500pc Rigaku diffractometer (CuK α radiation, working voltage 40 kV, working current 100 mA, current density 100 mA, Japan Science Corporation, Tokyo, Japan). The morphology of the catalyst was characterized by field emission scanning electron microscopy (FE-SEM; S-4800, Hitachi, Japan) and transmission electron microscopy (TEM) (JEOL JEM-2010, Japan Electronics Corporation, Tokyo, Japan). A UV-vis diffuse reflectance spectrometer (UV1901, Puxi, Beijing, China) was used for optical absorption measurement. Three-electrode quartz cells and a CHI660E electrochemical workstation (Shanghai Chenhua Co., Ltd., Shanghai, China) were used to test the electrochemical properties of the samples-with Pt as the counter electrode, saturated calomel electrode as the reference electrode, and 0.1M Na_2_SO_4_ as the electrolyte. Photoluminescence (PL) spectra were recorded on a F-7000 spectrometer (Hitachi, Japan). Gas chromatography for the determination of hydrogen in the reaction (purchased from Shimadzu Corporation, Kyoto, Japan). Freeze dryer for drying hydrogel (FD-1B-50, Shanghai Billion Instrument Manufacturing Co., Ltd., Shanghai, China).

## 3. Results and Discussion

### 3.1. Characterization of CdS NRs-SiO_2_HydrogelStructure

**Figure 1:**
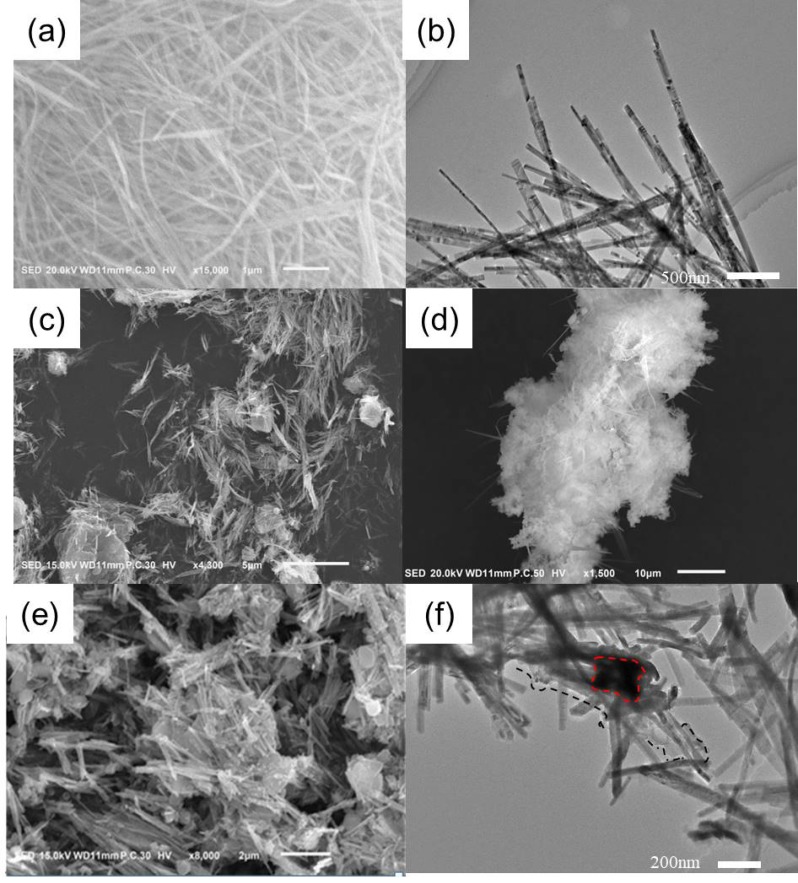
SEM of CdS NRs (**a**); CdS NRs-PANI (**c**) and CdS NRs-PANI-SiO_2_ hydrogel (**d**,**e**); TEM of CdS NRs (**b**) and CdS NRs-PANI-SiO_2_ hydrogel (**f**).

The morphology of the photocatalyst is characterized by TEM and SEM (Figure 1). Figure 1a,b shows SEM and TEM of CdS NRs, the diameter of nanorods was relatively uniform at about 20 nm and the length was about 2–3 μm. After being combined with PANI, as seen in Figure 1c, the morphology of CdS NRs was not visibly affected and they were covered with sheets of PANI. Figure 1d,e shows that CdS NRs-PANI was uniformly coated with SiO_2_ and formed into a three-dimensional network structure by cross-linking of SiO_2_ nanospheres. Figure 1f was a TEM diagram of ternary complexes. It can be seen that CdS NRs, PANI, and SiO_2_ were successfully linked together to form a 3D network structure.

**Figure 2:**
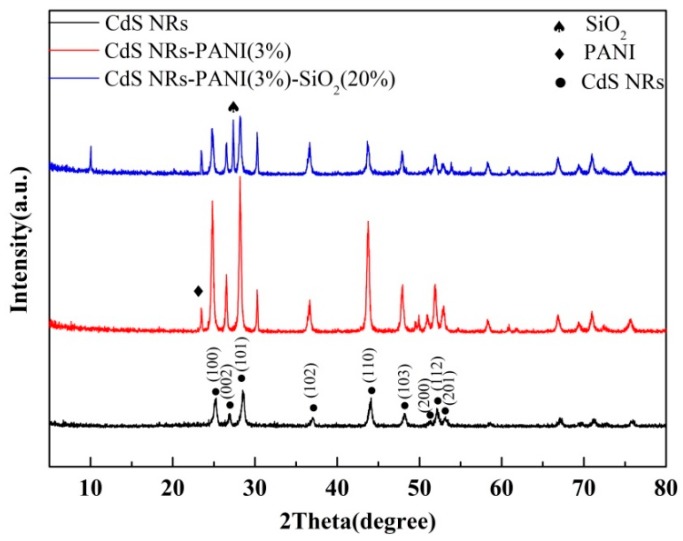
X-ray diffraction (XRD) spectra of CdS NRs, CdS NRs-PANI (3%), and CdS NRs-PANI (3%)-SiO_2_ (20%).

XRD was used to study the crystal structure of catalysts. The XRD data of CdS NRs, CdS NRs-PANI (3%) and CdS NRs-PANI (3%)-SiO_2_ (20%) are shown in Figure 2. From the data, it can be seen that the diffraction peaks of CdS NRs corresponding to (100), (002), (101), (110), (103), (200), (200) appeared, among which the (112) and (201) crystal planes were consistent with those reported in the literature [28,29]. The data also showed that CdS NRs was hexagonal and there were no characteristic peaks of other substances in its diffraction pattern. PANI mainly exists in an amorphous form. The diffraction peak at 24.6° was the characteristic peak of polyaniline which indicated that CdS NRs and polyaniline were successfully compounded. The diffraction peak at 27.32° belongs to the (110) plane of SiO_2_. The XRD diagram corresponding to CdS NRs-PANI (3%)-SiO_2_ (20%) complex showed similar diffraction peaks corresponding to CdS NRs-PANI (3%) but had lower intensity and a wider peak shape, which may be due to the amorphous nature of SiO_2_.

**Figure 3:**
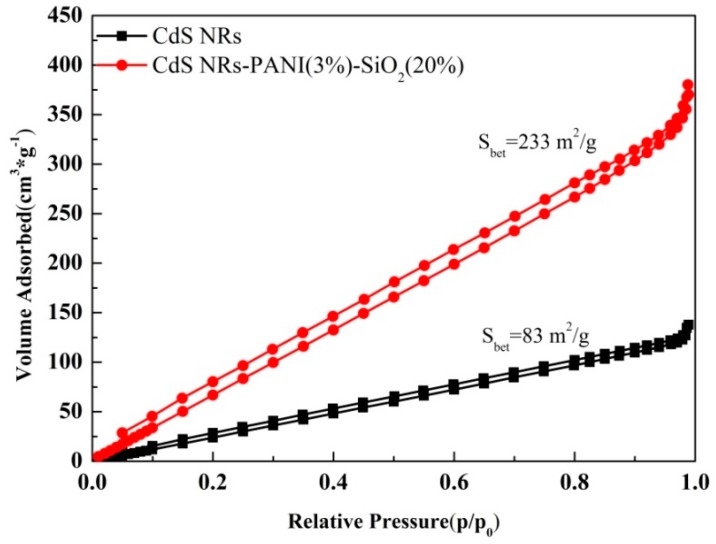
N_2_ adsorption–desorption isotherms of the catalysts.

In order to compare the specific surface area of composites with pure CdS NRs, the N_2_ adsorption–desorption isotherms were shown in Figure 3. The surface area of CdS NRs-PANI (3%)-SiO_2_ (20%) composites was much higher than that of CdS NRs, which would increase the active sites on the catalyst surface and promote hydrogen production during photocatalytic reaction.

### 3.2. Photoelectrochemical and Light Absorption Properties

**Figure 4:**
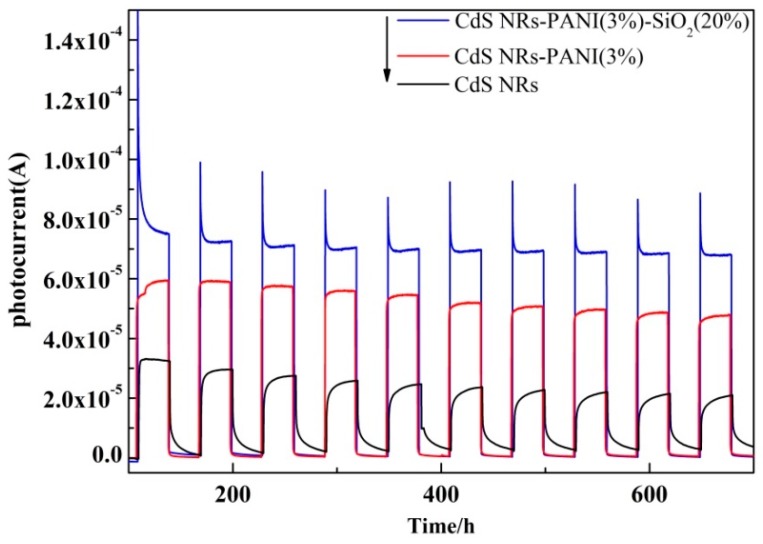
Transient photocurrent response of CdSNRs, CdS NRs-PANI (3%), and CdS NRs-PANI (3%)-SiO_2_ (20%).

To explore the electron–hole separation efficiency, the transient photocurrent response spectra of the photocatalyst [30] were tested under visible light irradiation. It was obvious from the spectra (Figure 4) that the photocurrent response of composite CdS NRs-PANI (3%)-SiO_2_ (20%) was significantly stronger than that of CdS NRs and CdS NRs-PANI (3%) under the same measurement conditions. Its photocurrent was increased 1.67 times and 3.19 times, respectively, compared with CdS NRs-PANI (3%) and CdS NRs. It is well known that high photocurrent corresponds to good conduction of photogenerated carriers [31,32]. The introduction of PANI and SiO_2_ played a significant role in promoting charge separation and transmission efficiency, improving the electrochemical performance of CdS NRs, and thus enhancing the catalytic activity of CdS NRs. As a good electron acceptor material, PANI could effectively transmit the CdS NRs excited electron, leading to more effective charge separation and migration on the catalyst surface. The addition of SiO_2_ gel reduced the aggregation of CdS NRs and indirectly inhibited the photoetching of CdS NRs, thereby improving the photoelectric properties of the catalyst.

**Figure 5:**
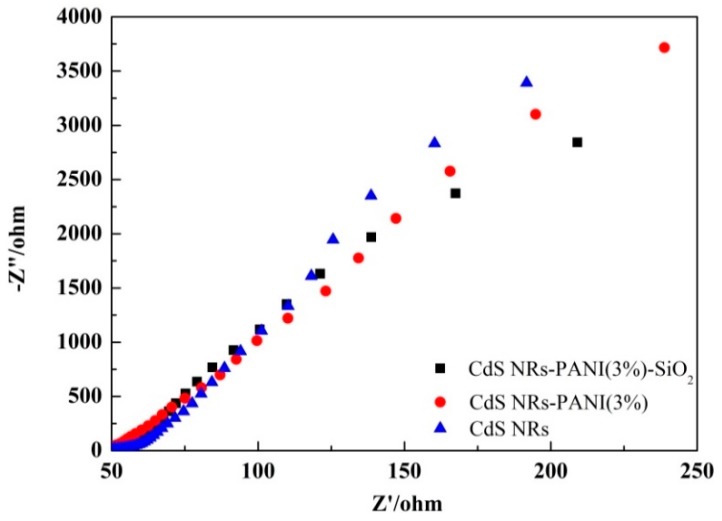
Electrochemical impedance spectra (EIS) plots of CdSNRs, CdS NRs-PANI (3%), and CdS NRs-PANI (3%)-SiO_2_ (20%).

In order to further prove that the electrical properties of the composites have been improved, the electrochemical impedance spectra (EIS) of CdS NRs, CdS NRs-PANI (3%), and CdS NRs-PANI (3%)-SiO_2_ (20%) were tested (Figure 5). The high frequency semicircle is a characteristic of the charge transfer process. The diameter of the semicircle is equal to the charge transfer resistance [33]. The Nyquist curve radius of CdS NRs-PANI (3%) and SiO_2_ (20%) ternary composite catalysts were lower than that of CdS NRs and CdS NRs-PANI (3%), indicating that the charge transfer resistance was the smallest and the surface electrode reaction rate was the largest. This was due to the uniform distribution of CdS NRs in SiO_2_ hydrogels, which reduced the aggregation of CdS NRs and increased the active sites of the complexes. At the same time, polyaniline can effectively promote electron transfer and improve the charge separation efficiency.The above results all indicate that the combination of PANI and SiO_2_ hydrogels of CdS NRs can effectively inhibit the recombination of the photoinduced electron–hole pairs and transmit more electrons to react on the surface.

**Figure 6:**
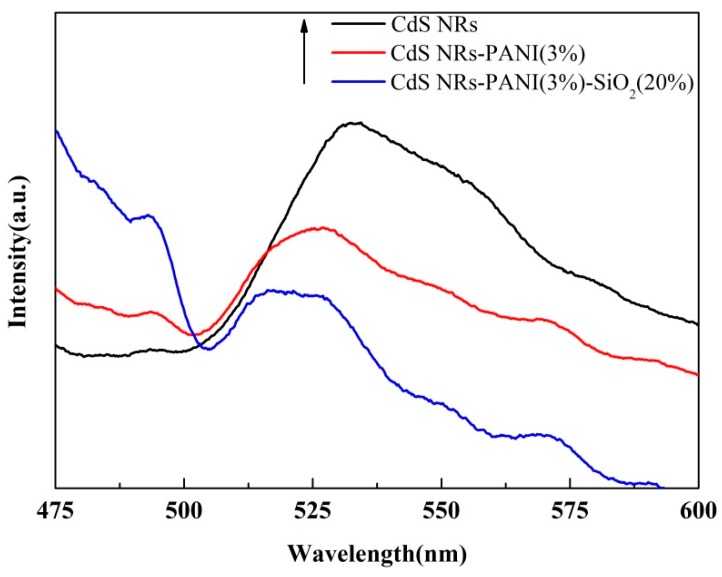
Photoluminescence spectra of CdS NRs, CdS NRs-PANI (3%), and CdS NRs-PANI (3%)-SiO_2_ (20%).

The recombination efficiency of photogenerated charge can also be analyzed by fluorescence spectroscopy [34,35]. Figure 6 shows the fluorescence spectra of CdS NRs, CdS NRs-PANI (3%), and CdS NRs-PANI (3%)-SiO_2_ (20%). There was a strong fluorescence peak [36] at 525 nm for CdS NRs monomers, caused mainly by electron–hole recombination. It can be seen that after the combination with PANI, the position of the peak did not change, but the intensity of the peak decreased gradually. After adding SiO_2_, the fluorescence peak became weaker. It indicated that the presence of PANI greatly inhibited the charge complexation and effectively improved the lifetime of photogenerated electrons and holes. The addition of SiO_2_ increased the specific surface area of the catalyst, which was also conducive to electron conduction. This result was consistent with the EIS and photocurrent experiments.

**Figure 7:**
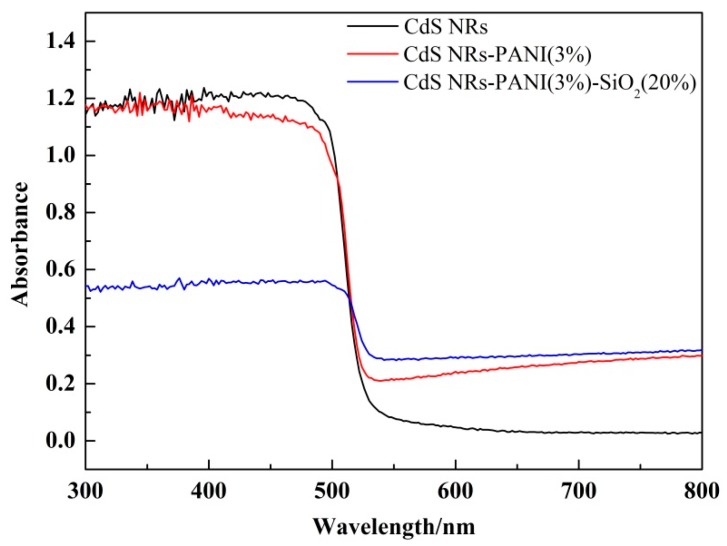
UV-visible diffuse reflectance spectra of CdS NRs, CdS NRs-PANI (3%), and CdS NRs-PANI (3%)-SiO_2_ (20%).

Figure 7 shows the UV-vis diffuse reflectance spectroscopy (DRS). Absorbance range increased gradually after adding PANI and SiO_2_, indicating that the optical absorption characteristics of CdS NRs-PANI and CdS NRs-PANI (3%)-SiO_2_ (20%) would be enhanced well after compounding and the utilization of visible light can be improved to a certain extent.

**Figure 8:**
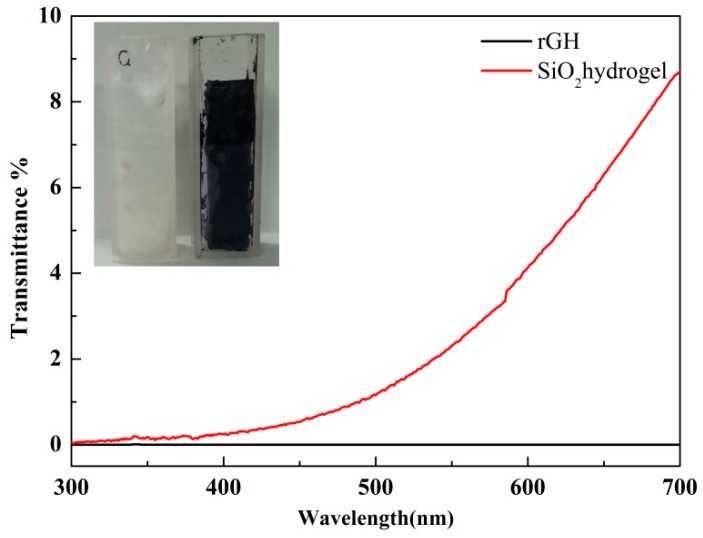
Light transmittance of SiO_2_ hydrogel and rGH.

The use of SiO_2_ hydrogel as a supporting substrate to develop a photocatalyst with a 3D network structure has no shading effect for the resultant photocatalyst compared with other common materials such as reduced graphene oxide hydrogel (rGH) [37,38]. Figure 8 showed the transmittance test chart of rGH and SiO_2_ gel. It can be seen from the diagram that the transmittance of SiO_2_ gel under visible light was obviously higher than that of rGH, indicating that the use of SiO_2_ gel effectively avoided the shortcoming of easy shading.

### 3.3. The Photocatalytic Hydrogen Production Performance of 3D Network CdS NRs-PANI-SiO_2_ Hydrogel

**Figure 9:**
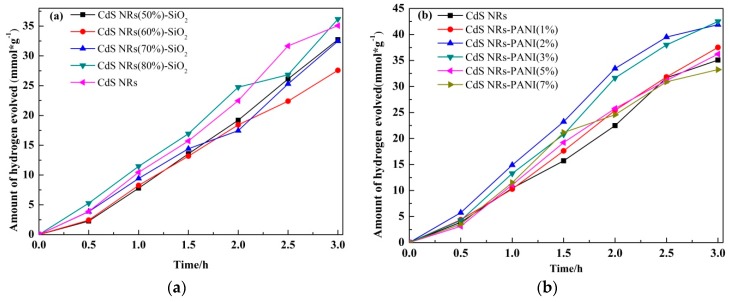
Photocatalytic hydrogen production activities of CdS NRs-SiO_2_ hydrogel with different proportions (**a**) and CdS-PANI binary composites with different ratios (**b**).

The hydrogen evolution activities of different samples were investigated under visible light irradiation. When CdS NRs was combined with SiO_2_to form two composite hydrogels, it can be seen from Figure 9a that the photocatalytic hydrogen evolution activity of CdS NRs (80%)-SiO_2_ was the highest with 36.16 mmol/g in 3 h, and as the amount of CdS NRs content increased, the photocatalytic activity of hydrogen production was increased. It was found that the hydrogen production rate of CdS NRs (80%)-SiO_2_ reached 12.05 mmol·g^−^^1^·h^−^^1^ and its photocatalytic activity was higher than pure CdS NRs. This may be due to the addition of SiO_2_, which could reduce the agglomeration of the reagent and increase the active sites on the surface. In Figure 9b, the effect of PANI content on the activity of catalyst CdS NRs was investigated. The activity of composite materials with different PANI content was tested. It can be seen that with the increase of PANI content, the activity of photocatalytic hydrogen production increased gradually. When the amount of PANI was 3 wt %, the activity of photocatalytic hydrogen production was the highest. The amount of hydrogen production reached 42.5 mmol/g in 3 h and the rate of hydrogen production was 14.16 mmol·g^−1^·h^−1^. Compared with the monomer CdS NRs, the catalytic activity decreased when the content reached 5 wt % and after that the activity decreased gradually with the increase of the proportion of PANI. From these data, we can intuitively observe that the amount of PANI composite significantly affected the photocatalytic hydrogen production capacity of the composite. This suggested that the addition of PANI promoted the activity of CdS NRs in a certain range.

**Figure 10:**
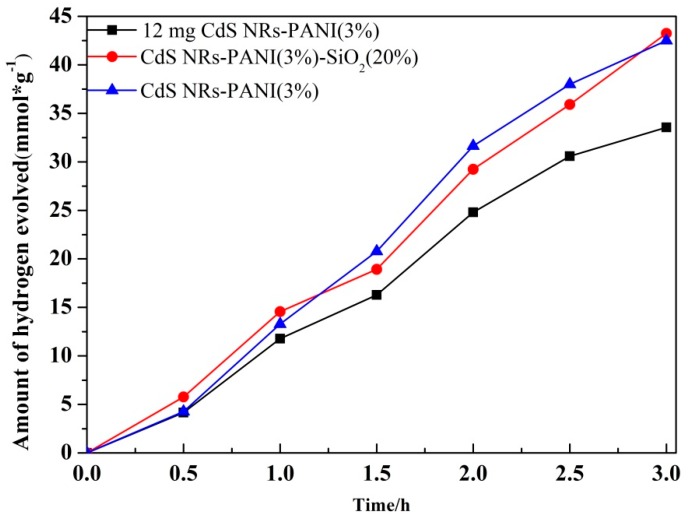
Photocatalytic hydrogen production activity of CdS NRs-PANI (3%)-SiO_2_ (20%).

As shown in Figure 10, it was observed that the hydrogen production amount of the ternary composite CdS NRs-PANI (3%)-SiO_2_ (20%) was as high as 43.25 mmol/g. Compared with the equivalent CdS in CdS NRs-PANI (3%), the photocatalytic activity of hydrogen production increased obviously. It may be due to SiO_2_ gel to increase the specific surface area of the catalyst and the active sites of the catalyst surface. Compared with the same amount of CdS NRs-PANI (3%) as the catalyst, the hydrogen evolution activity of the catalyst was improved slightly. This was due to the fact that with the increase of SiO_2_ content, the content of CdS NRs decreased, meaning the photocatalytic hydrogen production activity was not significantly improved.

**Figure 11:**
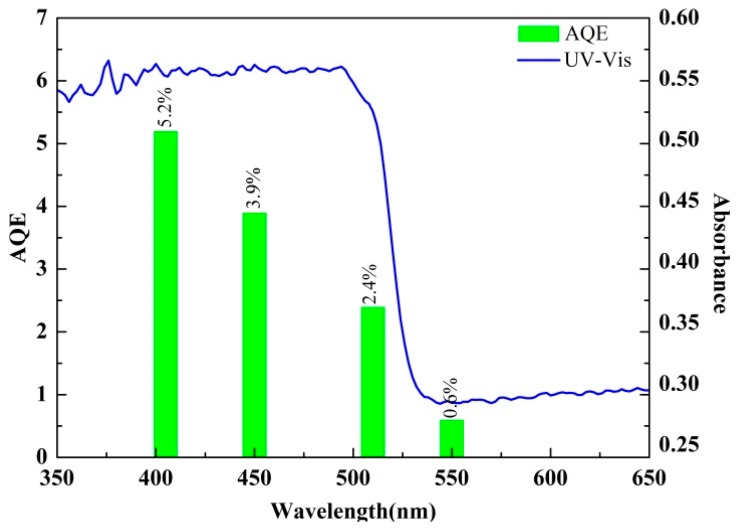
AQE of CdS NRs-PANI (3%)-SiO_2_ (20%) at different wavelengths.

Figure 11 shows the activity diagram of photocatalytic hydrogen production under different visible light wavelengths (405, 450, 510, and 550 nm). The AQE of the catalyst reached 5.2% at 405 nm and, with the increase of the wavelength, the AQE decreased. Under the condition of 550 nm, the AQY of the composite catalyst decreased to 0.6%. This result was consistent with the optical properties obtained by experiments. The optical absorption characteristics of the catalyst had a significant effect on the activity of the catalyst.

**Figure 12:**
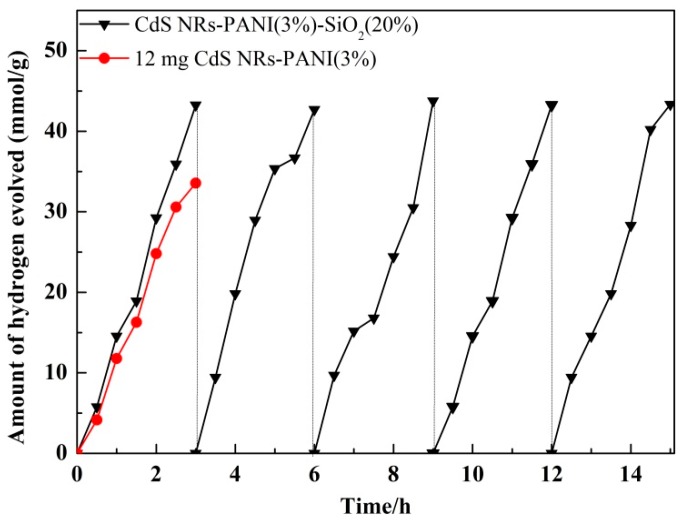
CdS NRs-PANI (3%)-SiO_2_ (20%) photo-catalytic hydrogen production cycle test.

The cyclic stability of CdS NRs-PANI (3%)-SiO_2_ (20%) for photocatalytic hydrogen production was tested and the results are shown in Figure 12. It was observed that the activity of the composite catalyst remained unchanged after five cycles. The reduction of the electron–hole pair recombination, due to the addition of polyaniline, effectively inhibited the light corrosion of CdS NRs. Furthermore, the addition of SiO_2_ gel increased the specific surface area of the catalyst and exposed more active sites, which were beneficial for the transport of electrons and improving the photocatalytic stability of the catalyst as a whole.

Table 1 shows the pore size test data of different catalysts. The interception rate of CdS NRs-PANI (3%)-SiO_2_ (20%) was 91.3% when passing through a 25 μm aperture, but the interception rate of CdS NRs was only 45%. As seen in Table 1, mixed hydrogels can be recovered through stainless-steel filters. In the absence of additional pressure, the recovery rate can reach more than 90%, which reduced energy consumption and catalyst recovery costs.

### 3.4. Study of the Photocatalytic Mechanism

**Figure 13:**
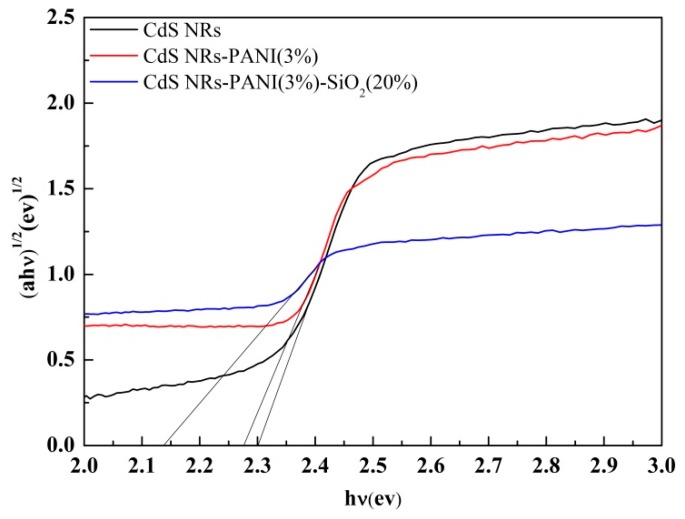
Kubelka–Munk conversion curve of CdS NRs, CdS NRs-PANI (3%), and CdS NRs-PANI (3%)-SiO_2_ (20%).

The Kubelka–Munk equation can be used to calculate the band gap of semiconductors. According to the value of CdS [39,40] and Kubelka–Munk conversion curve in Figure 13, the band gap of CdS NRs, binary, and ternary complexes were estimated to be 2.3 eV, 2.28 eV, and 2.15 eV, respectively, which corresponded to the results of ultraviolet spectroscopy. Compared with CdS NRs, the band gap of ternary composites was narrower, which was beneficial to the absorption of visible light, thus improving the photocatalytic performance.

**Figure 14:**
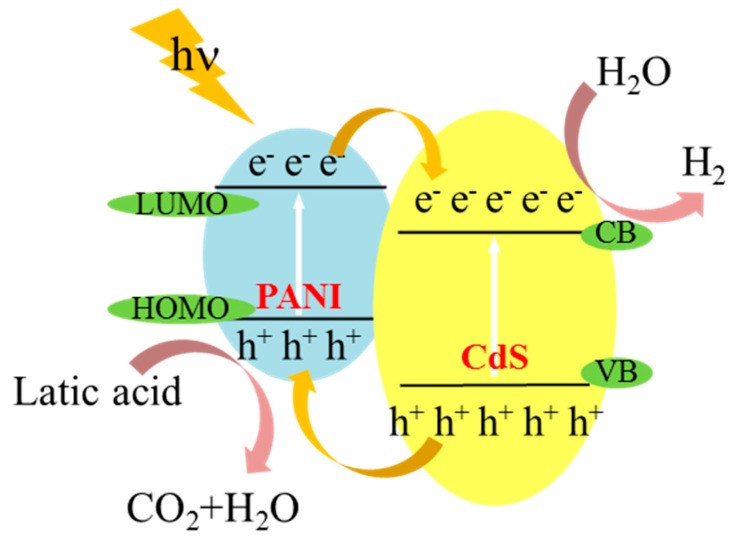
CdS NRs-PANI-SiO_2_ hydrogel photo-catalytic hydrogen production mechanism under visible light.

According to the above results, the photocatalytic mechanism was speculated as shown in Figure 14. Under visible light irradiation, electrons on both CdS NRs and PANI could be excited. From the energy band position, CdS NRs had a more positive energy level position than PANI, so electrons on the PANI monomer LUMO were easier to transfer to CdS NRs. On the other hand, the holes in the VB orbits of CdS NRs were easier to transfer to the HOMO orbits of PANI, and the photogenerated electrons would be rapidly transported to the surface of CdS NRs to realize the effective separation of e^−^ and h^+^. Electrons on the surface of CdS NRs were reduced to form hydrogen. The matching of energy levels made it easier for CdS NRs-PANI composites to achieve effective separation of photogenerated e^−^/h^+^ pairs and improved the lifetime of photogenerated charges. The three-dimensional structure of SiO_2_ gel reduced the aggregation of CdS NRs, which improved the specific surface area of the catalyst and increased the active sites on the catalyst surface, resulting in effective photocatalytic performance.

## 4. Conclusions

In summary, the conjugated PANI nanosheets were covered on the surface of CdS NRs and the photocatalytic hydrogen production activity of the CdS NRs-PANI composite was significantly improved. Furthermore, SiO_2_ gel was exploited to construct the 3D network structure of CdS NRs-PANI-SiO_2_. Through a series of characterization tests and activity tests of the catalyst, it was shown that PANI can effectively promote the transport rate of photogenerated carriers. The addition of SiO_2_ gel was also beneficial to the catalyst to expose more active sites. Meanwhile, the experimental results showed that the hydrogen production amount under visible light was 1.3 times that of CdS NRs. Importantly, the hydrogen production efficiency of the catalyst remained unchanged after five cycles indicating its strong stability and the photocatalyst was also easy to recycle.

## Figures and Tables

**Table 1:** Pore retention of different materials.

Filter (Aperture)	25 μm	38 μm	75 μm
CdS NRs-PANI (3%)-SiO_2_ (20%)	91.3%	70.8%	29%
CdS NRs	45%	30%	8%
